# Resection of Four Feet of Intestines

**Published:** 1901-06

**Authors:** 


### Resection of Four Feet of Intestines.

Dr. G. D. McNutt reports an unusually interesting case of abdominal surgery. In December, 1896, he was called to see a woman, sixty-two years old, who had been suffering four days with the symptoms of complete bowel obstruction. A small strangulated femoral hernia was discovered. The abdomen was opened and four inches of the bowel removed. The patient made a good recovery and remained in perfect health until April, 1899, at which time a considerable length of the small intestine was strangulated by being forced through a hole in the omentum. After suffering for eight days in this condition she submitted to an operation. It was found necessary to remove forty-eight inches of the small bowel. The ends were united by a Murphy button, which was passed on the tenth day. The patient made a good recovery and is today in fair health.—*Pennsylvania Medical Journal,* April, 1901.

